# The effect of positive end-expiratory pressure on intracranial pressure in obese and non-obese severe brain injury patients: a retrospective observational study

**DOI:** 10.1186/s12871-022-01934-9

**Published:** 2022-12-15

**Authors:** Dawei Zhou, Tong Li, Shuyang Fei, Chao Wang, Yi Lv

**Affiliations:** Department of Critical Care Medicine, Beijing Tongren Hospital, Capital Medical University, Beijing, China

**Keywords:** Intracranial pressure, Positive end-expiratory pressure, Cerebral perfusion pressure, Obesity, Severe brain injury

## Abstract

**Background:**

The effect of positive end-expiratory pressure (PEEP) on intracranial pressure (ICP) had never been studied in obese patients with severe brain injury (SBI). The main aim was to evaluate the effect of PEEP on ICP in SBI patients with mechanical ventilation according to obesity status.

**Methods:**

SBI patients admitted to the ICU with mechanical ventilation between 2014 and 2015 were included. Demographic, hemodynamic, arterial blood gas, and ventilator data at the time of the paired PEEP and ICP observations were recorded and compared between obese (body mass index ≥ 30 kg/m^2^) and non-obese SBI patients. Generalized estimating equation (GEE) model was used to assess the relationship between PEEP and ICP in obese and non-obese SBI patients, respectively.

**Results:**

Six hundred twenty-seven SBI patients were included, 407 (65%) non-obese and 220 (35%) obese patients. A total of 30,415 paired PEEP and ICP observations were recorded in these patients, 19,566 (64.3%) for non-obese and 10,849 (35.7%) for obese. In the multivariable analysis, a statistically significant relationship between PEEP and ICP was found in obese SBI patients, but not in non-obese ones. For every cmH_2_O increase in PEEP, there was a 0.19 mmHg increase in ICP (95% CI [0.05, 0.33], *P* = 0.007) and a 0.15 mmHg decrease in CPP (95% CI [-0.29, -0.01], *P* = 0.036) in obese SBI patients after adjusting for confounders.

**Conclusions:**

The results suggested that, contrary to non-obese SBI patients, the application of PEEP may produce an increase in ICP in obese SBI patients. However, the effect was modest and may be clinically inconsequential.

## Introduction

Obesity is defined as the condition of excess or abnormal fat accumulation which could increase risks to health by World Health Organization (WHO) [1]. The overall prevalence of obesity is rising worldwide and as well as in the intensive care unit (ICU) [2, 3]. Obesity is considered as one of the specific characteristics for ICU patients, especially regarding respiratory care [1, 4, 5]. For obese patients, the respiratory system, especially the chest wall, becomes ‘stiffer’ [6–8].

Acute lung injury (ALI) is one of the common complications for patients with severe brain injury (SBI). The incidence has been reported to be 5 to 30% [9]. Mechanical ventilation is the main supportive therapy for ALI with the aim to supply sufficient oxygen and remove carbon dioxide produced by the peripheral organs [10, 11]. For severe ALI, high positive end-expiratory pressure (PEEP) may be needed [12]. However, previous studies showed that PEEP may have an influence on intracranial pressure (ICP) [13]. The relationship between PEEP and ICP had been investigated for decades; however, no consensus had been reached [14].

Previous studies showed that several factors were associated with the effect of PEEP on ICP, including lung recruitability, chest wall elastance, respiratory mechanics, intracranial compliance, and baseline ICP [14]. Among these factors, the respiratory mechanics may play an important role [15–17]. The transmission of PEEP to pleural space depends on the elastance of both the lungs and chest wall [18]. It seemed that the stiffer chest wall had higher pressure transmission [19, 20].

Previous studies reported that obese patients had higher chest wall elastance [7, 8]. Given the specific pathophysiology for obese patients, the obesity status could be a confounding factor for the relationship of PEEP and ICP. The aim of this study was to determine the effect of obesity status on the relationship of PEEP and ICP for patients with SBI. We hypothesized that the application of PEEP may produce different effect on ICP for obese and non-obese SBI patients.

## Methods

### Setting

The eICU (eicu-crd.mit.edu) database was used in the present study, which included patients at 208 hospitals located throughout the US admitted between 2014 and 2015. The dedicated description of eICU database is available elsewhere [21]. The database was exempted from institutional review board approval due to the retrospective design, lack of direct patient intervention, and the security schema. Researchers were allowed to access to the database under reasonable requirement.

### Study population

All patients in the eICU were eligible for inclusion in the present investigation. As for those who admitted to ICU for more than once, only the first ICU stay was included. Acute SBI (including traumatic brain injury, intracranial hemorrhage, subarachnoid hemorrhage, ischemic stroke, brain tumor, subdural hematoma, and others, with Glasgow Coma Score [GCS] < 9) adult patients who received ICP monitoring and invasive mechanical ventilation at the same time were selected. Patients with incomplete weight and height data and inconsistent time of ICP and PEEP recorded were excluded.

### Clinical variables and outcomes

Data were extracted on the following information during the first 24 h of ICU admission: age, gender, height, weight, ethnicity (Caucasian, African American, Hispanic, Native American, other/unknown), admission diagnosis, GCS and Acute Physiology and Chronic Health Evaluation (APACHE) IV score. Therapeutic drugs including mannitol, hypertonic saline, and vasopressors during the entire ICU stay was collected. Outcomes including time of invasive mechanical ventilation, ICU length of stay, ICU mortality, and hospital mortality were also extracted. ICP monitoring time was the difference of the time between the last and the first recording. In accordance with international standards, patients with a body mass index (BMI) ≥ 30 kg/(m^2^) were defined as obese [22], where BMI was calculated as body weight /(height^2^).

Clinical parameters, including parameters of mechanical ventilation [PEEP, plateau pressure, respiratory rate, tidal volume, tidal volume to predicted body weight [23], and the fraction of inspired oxygen (FiO_2_)], arterial blood gas (ABG), and hemodynamic parameters were time-stamped variables, both time and value were recorded. ABG parameters included arterial partial pressure of carbon dioxide (PaCO_2_), partial pressure of oxygen (PaO_2_), pH, and the ratio of the PaO_2_ and FiO_2_ (P/F). Hemodynamic parameters contained heart rate (HR), systolic blood pressure (BP), diastolic BP and mean arterial BP.

The severity of lung injury (LI) was defined by the PaO_2_/FiO_2_ as used by the Berlin criteria for acute respiratory distress syndrome (ARDS) [24]. Based on the criteria, P/F ratio > 300 was defined as no LI, mild LI was defined as P/F ratio > 200 and ≤300, moderate LI was P/F ratio > 100 and ≤200, and severe was P/F ratio ≤100. ICP was recorded and validated about 5 min at regular intervals in eICU database, and the mean ICP during one hour was calculated. The same method was used to calculate the cerebral perfusion pressure (CPP), which was difference between mean BP and ICP at the same time. The primary exposure of interest was the paired PEEP and ICP at the same hour. The primary outcome was the correlation of paired PEEP and ICP between obese and non-obese patients.

### Statistical analysis

Continuous variables were reported as mean and standard deviation (SD) or median and interquartile range (IQR), compared using Student’s t-test or Wilcoxon-rank-sum test according to normality test. Categorical variables were reported as numbers and percentages and were analyzed with Chi-square test or Fisher’s exact test as appropriate. The data were cleaned for completeness and consistency. The outliers outside 3×IQR, less than triple lower quartile or greater than triple upper quartile, were checked and substituted by the 5th or 95th percentile.

The descriptive analysis was firstly performed in non-obese and obese patients, including baseline characteristics and clinical parameters. Secondly, generalized estimating equations (GEE) models were used to assess the relationship between PEEP and ICP as well as PEEP and CPP. GEE was used to find the association between a repeated measure variable (PEEP) and an outcome variable (ICP). The autocorrelation was used as the correlation structure for GEE model. We classified the observation data by BMI category and severity of LI. The relationship between PEEP and ICP or CPP was assessed respectively with both univariable and multivariable analysis. A total of 14 subgroups including obese, non-obese, no lung injury, mild lung injury, moderate lung injury, severe lung injury, obese and no lung injury, obese and mild lung injury, obese and moderate lung injury, obese and severe lung injury, non-obese and no lung injury, non-obese and mild lung injury, non-obese and moderate lung jury, and non-obese and severe lung injury were investigated respectively. Variables with p values < 0.2 in the univariate analysis were entered into the multivariate GEE model. The confounders included age, GCS on admission, APACHE IV score without GCS score, tidal volume per PBW, respiratory rate, plateau pressure, mean BP, PaCO_2_ and usage of vasopressors, mannitol or hypertonic saline.

Data extraction was performed using PostgreSQL (www.postgresql.org). *P* value less than 0.05 was considered statistically significant. R software (4.0.1, www.r-project.org) was used for all the statistical analyses.

## Results

**Fig. 1:**
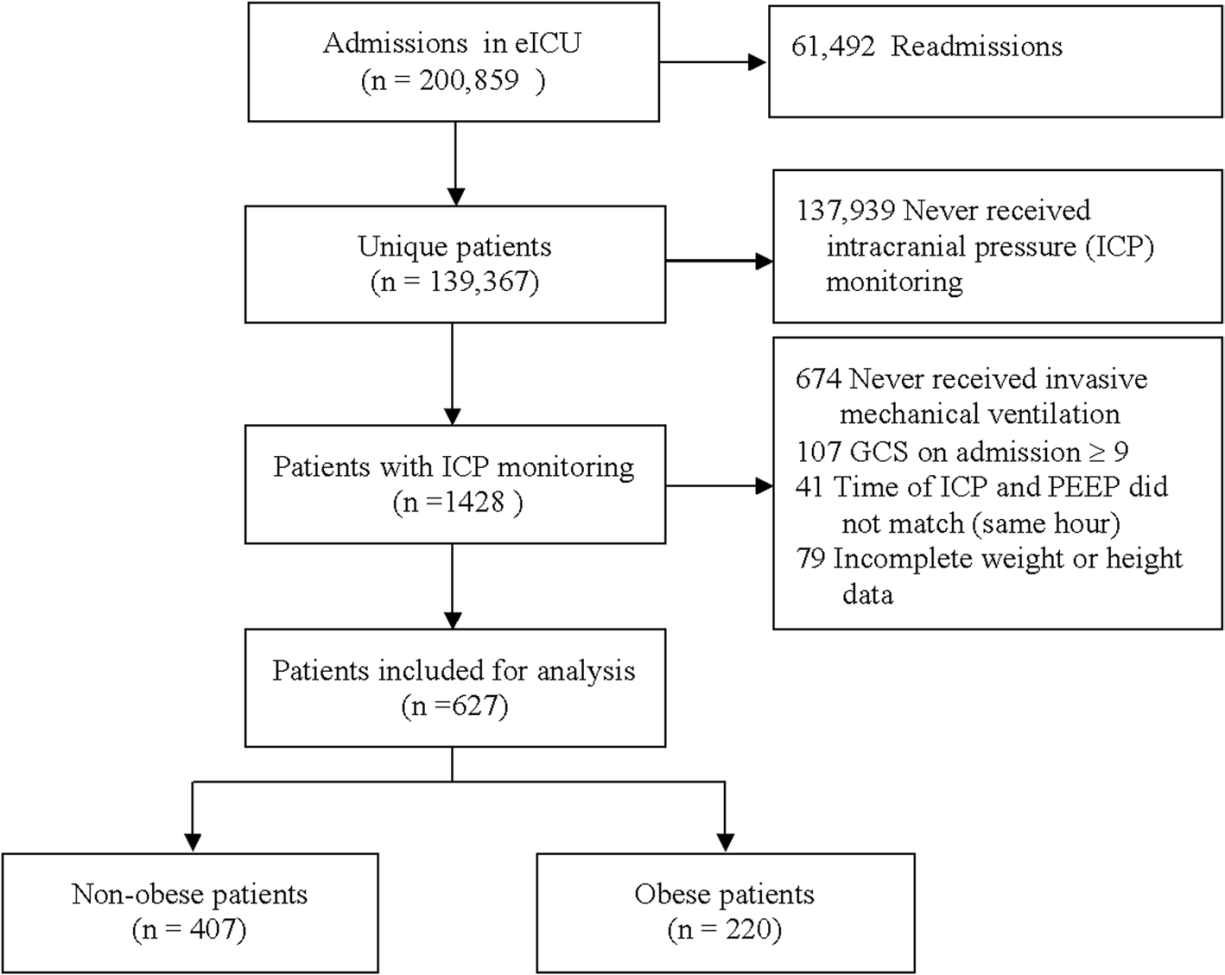
Flowchart of subject selection

Overall, the eICU database recorded 200,839 patient admissions and 139,367 unique patients. After exclusion, a total of 627 patients were included for analysis (Fig. 1), of which 220 (35%) were obese patients and 427 (65%) were non-obese patients. The mean age was 54 (IQR 38–66) years. The median BMI in the total population was 27 (IQR 34 − 32) kg/m^2^, 25 (IQR 22–27) kg/m^2^ for non-obese and 34 (IQR 32–38) kg/m^2^ for obese patients. Demographics and baseline characteristics between obese and non-obese patients were presented in Table 1. Non-obese patients had higher percentages of no lung injury (63% VS 44%) and a trend of higher percentages of traumatic brain injury (32% VS 23%, *P* = 0.07), while obese patients had higher percentage of subarachnoid hemorrhage (15% VS 12%) and ischemic stroke (10% VS 7%). The median invasive ventilation time for non-obese patients was 6 (IQR 3–12) days and obese patients was 7 (IQR 4–13) days. The median ICP monitoring time for non-obese patients was 3 (IQR 1–8) days and obese patients was 4 (IQR 1–8) days. In non-obese patients, the ICU and hospital mortality rates were 24% and 31% respectively, while for obese patients, were 28% and 38% respectively.

**Table 1:** Comparison of characteristics between obese and non-obese patients

	Total (*n* = 627)	Non-obese (*n* = 407)	Obese (*n* = 220)	*P* value
Age (years)	54 (38, 66)	54 (35, 66)	54 (44, 65)	0.355
Gender (male)	389 (62)	247 (61)	142 (65)	0.388
Weight (kg)	80 (68, 98)	73 (63, 80)	105 (93, 120)	< 0.001
Height (cm)	173 (165, 180)	171 (165, 180)	173 (165, 183)	0.035
BMI (kg/m^2^)	27 (24, 32)	25 (22, 27)	34 (32, 38)	< 0.001
Ethnicity				0.071
Caucasian	457 (73)	287 (71)	170 (77)	
African American	71 (11)	47 (12)	24 (11)	
Hispanic	27 (4)	19 (5)	8 (4)	
Native American	8 (1)	4 (1)	4 (2)	
Other/Unknown	64 (11)	50 (13)	14 (6)	
Lung Injury				< 0.001
No	352 (56)	256 (63)	96 (44)	
Mild	118 (19)	70 (17)	48 (22)	
Moderate	119 (19)	58 (14)	61 (27)	
Severe	38 (6)	23 (6)	15 (7)	
Cause of brain injury				0.07
Traumatic brain injury	180 (29)	129 (32)	51 (23)	
Intracranial Hemorrhage	169 (27)	114 (28)	55 (25)	
Subarachnoid Hemorrhage	81 (13)	48 (12)	33 (15)	
Ischemic Stroke	51 (8)	29 (7)	22 (10)	
Brain tumor	30 (5)	16 (4)	14 (6)	
Subdural Hematoma	24 (4)	18 (4)	6 (3)	
Others	82 (15)	53 (13)	39 (18)	
Categories of traumatic brain injury (*n* = 180)				0.984
Head/only	66 (11)	47 (12)	19 (9)	
Head/multiple	70 (11)	50 (12)	20 (9)	
Head/other	44 (7)	32 (8)	12 (5)	
GCS on admission	6 (3, 7)	6 (3, 7)	5 (3, 7)	0.791
APACHE IV score	78 (63, 93)	78 (63, 92)	79 (64, 96)	0.491
Invasive ventilation time (days)	7 (3, 13)	6 (3, 12)	7 (4, 13)	0.317
ICP monitoring time (days)	3 (1, 8)	3 (1, 8)	4 (1, 8)	0.535
Hypertonic saline	116 (19)	74 (18)	42 (19)	0.863
Mannitol	121 (19)	79 (19)	42 (19)	0.998
Vasopressors	328 (52)	212 (52)	116 (53)	0.945
ICU length of stay (hours)	250 (106, 424)	247 (105, 421)	253 (116, 425)	0.79
ICU mortality	158 (25)	96 (24)	62 (28)	0.243
Hospital mortality	210 (33)	126 (31)	84 (38)	0.082

Data are median (interquartile range) or no./total (%)

APACHE IV: Acute Physiology and Chronic Health Evaluation IV, BMI: body mass index, GCS: Glasgow Coma Scale, ICP: intracranial pressure, ICU: intensive care unit

The characteristics of clinical parameters between obese and non-obese patients including mechanical ventilation and hemodynamic data paired to each ICP observation time were displayed in Table 2. Of the 30,415 observations (paired PEEP and ICP), 19,566 (64.3%) were for non-obese patients, and 10,849 (35.7%) for obese patients. Compared with non-obese patients, the obese group had higher ICP (10 vs. 9 mmHg, *P* < 0.001), higher plateau pressure (19 vs. 17 mmHg, *P* < 0.001), high tidal volume (520 vs. 500 ml, *P* < 0.001) and tidal volume per PBW (7.6 vs. 7.3, *P* < 0.001), lower P/F ratio (308 vs. 357, *P* < 0.001) and higher mean BP (87 vs. 86 mmHg, *P* < 0.001).

**Table 2:** Clinical parameters between non-obese and obese

	Total observations (*n* = 30,415)	Non-obese (*n* = 19,566)	Obese (*n* = 10,849)	*P* value
Cerebral parameters				
ICP (mmHg)	9 (5, 14)	9 (5, 13)	10 (6, 15)	< 0.001
CPP (mmHg)	76 (68, 85)	76 (68, 85)	76 (67, 85)	0.006
Ventilation parameters				
PEEP (cmH_2_O)	5 (5, 8)	5 (5, 8)	5 (5, 8)	< 0.001
Plateau Pressure (cmH_2_O)	18 (15, 20)	17 (15, 19)	19 (16, 22)	< 0.001
Tidal Volume (ml)	500 (450, 550)	500 (450, 550)	520 (475, 580)	< 0.001
Tidal Volume/PBW (ml/kg)	7.3 (6.5, 8.1)	7.3 (6.5, 8.0)	7.6 (6.8, 8.4)	< 0.001
Respiratory Rate (bpm)	18 (17, 21)	18 (16, 21)	18 (17, 21)	< 0.001
FiO_2_	40 (40, 49)	40 (40, 40)	40 (40, 50)	< 0.001
Laboratory parameters				
PaCO_2_ (mmHg)	36 (32, 40)	36 (31, 39)	37 (33, 41)	< 0.001
PaO_2_ (mmHg)	149 (104, 208)	159 (114,216)	137 (95, 172)	< 0.001
pH	7.39 (7.33, 7.43)	7.39 (7.33, 7.43)	7.39 (7.32, 7.43)	0.075
PaO_2_/FiO_2_	344 (212, 421)	357 (242, 421)	308 (180, 413)	< 0.001
Hemodynamic parameters				
Heart Rate (bpm)	83 (72, 95)	84 (72, 97)	82 (72, 93)	< 0.001
Systolic BP (mmHg)	132 (122, 145)	131 (121, 144)	135 (124, 147)	< 0.001
Diastolic BP (mmHg)	66 (58, 72)	65 (57, 72)	66 (59, 73)	< 0.001
Mean arterial BP (mmHg)	86 (79, 94)	86 (78, 93)	87 (79, 95)	< 0.001

Data are median (interquartile range) or no./total (%)

Each observation refers to a paired ICP and PEEP data point, not an individual patient

*BP* blood pressure, *bpm *beat per minute, *CPP* cerebral perfusion pressure, *FiO*_*2*_ fraction of inspired oxygen, *ICP *intracranial pressure, *PBW *predicted body weight, *PEEP *positive end-expiratory pressure

**Fig. 2:**
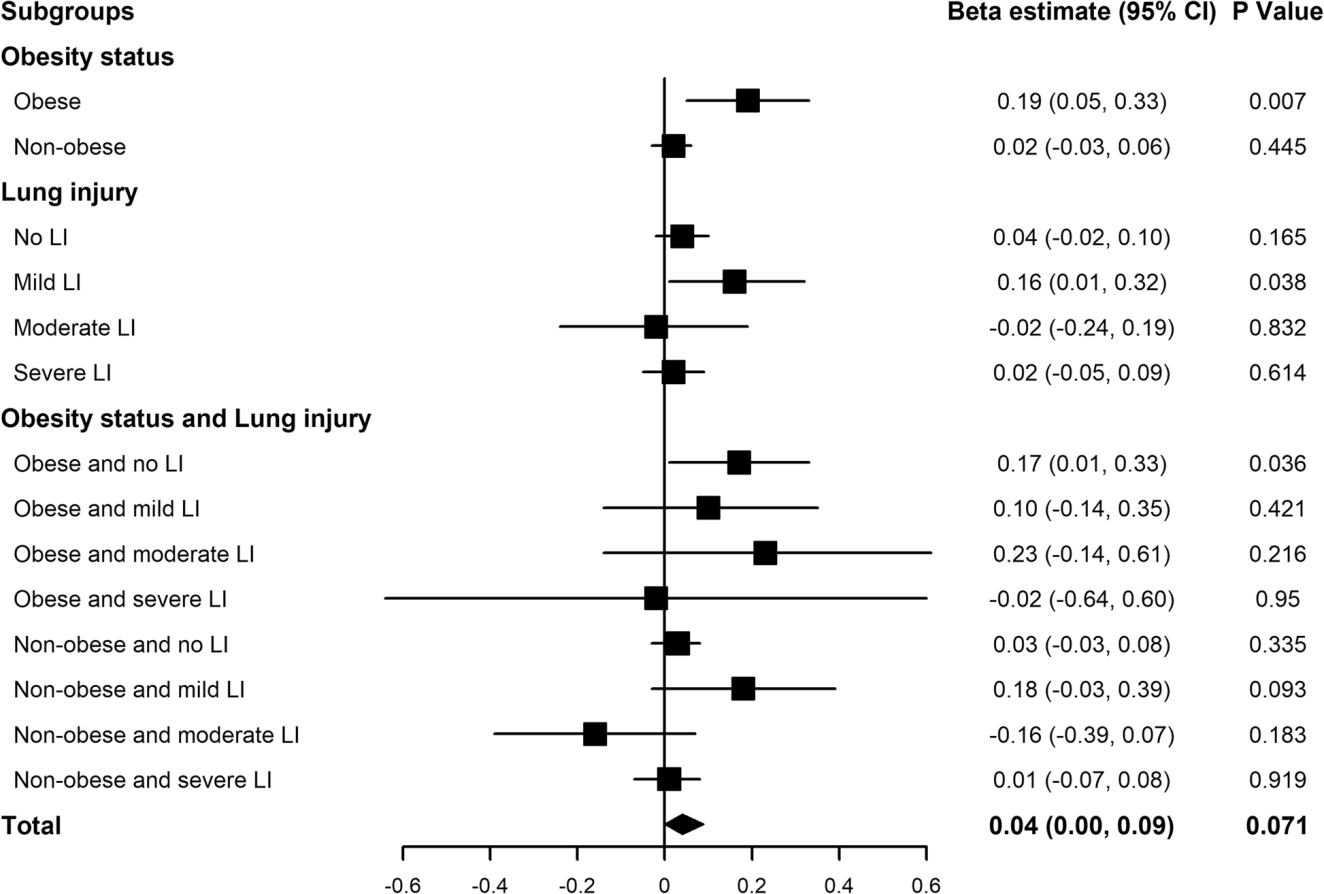
The beta estimate coefficient of effect of PEEP on ICP in multivariable GEE model in different subgroups. PEEP positive end-expiratory pressure, ICP intracranial pressure, GEE generalized estimating equation

**Fig. 3:**
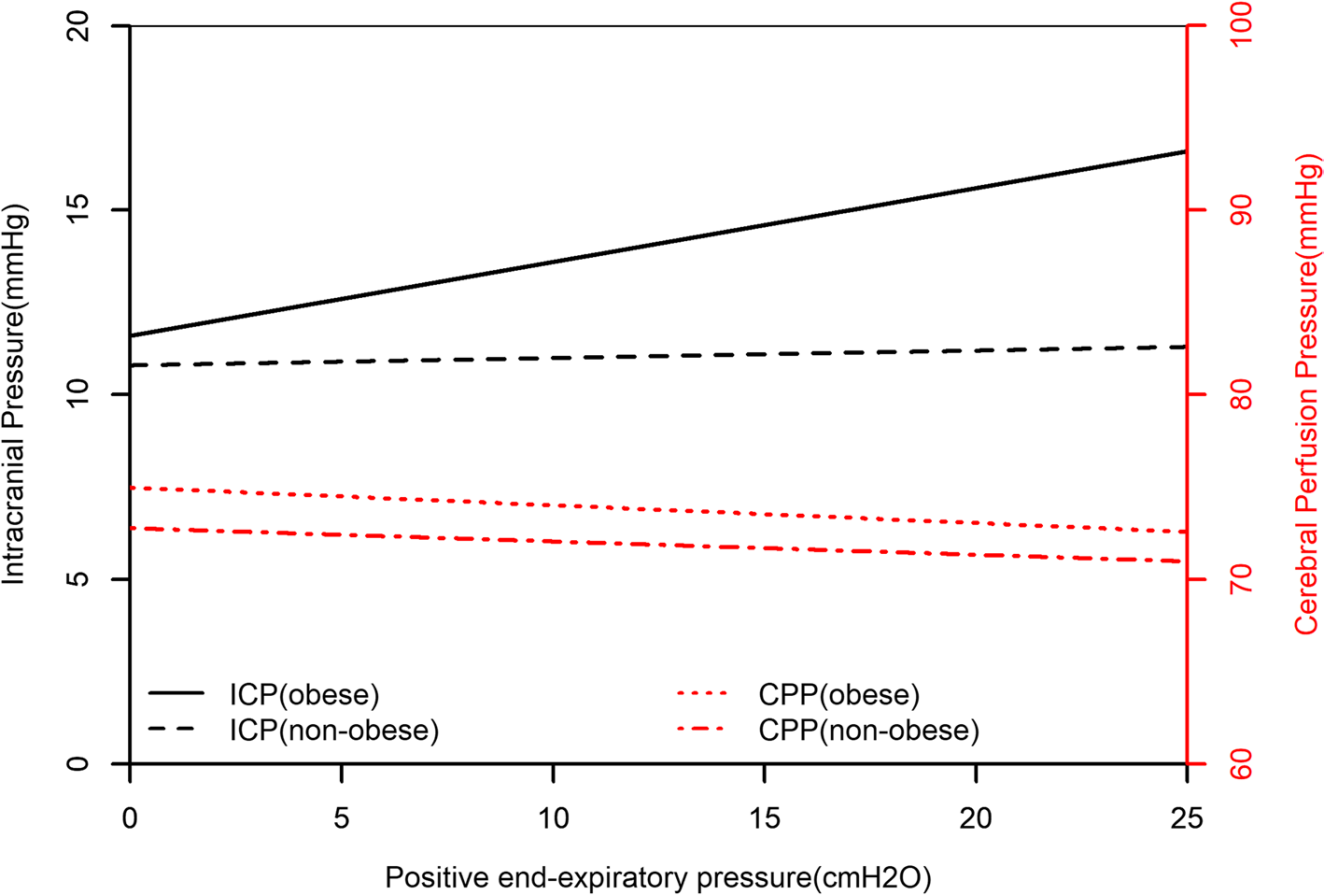
Univariate analysis of the relationship between PEEP and ICP or CPP in obese and non-obese groups (obese patients experienced an increase of ICP 0.2 mmHg (95% CI [0.06, 0.34], *P* = 0.004) with 1 cmH_2_O PEEP, while it was nonsignificant for others (non-obese patients experienced an increase of ICP 0.02 mmHg (95% CI [-0.03, 0.06], *P* = 0.423; obese patients experienced an increase of CPP − 0.12 mmHg (95% CI [-0.44, 0.2], *P* = 0.452; non-obese patients experienced an increase of CPP − 0.09 mmHg (95% CI [-0.21, 0.04], *P* = 0.193). ICP intracranial pressure, CPP cerebral perfusion pressure, CI confidence interval, PEEP positive end-expiratory pressure

Table 3 showed the unadjusted and adjusted analyses for the effect of PEEP on ICP and CPP stratified by different subgroups. Univariate analyses indicated that for every centimeter H_2_O (cm H_2_O) increase in PEEP, obese patients experienced an increase of 0.2 mmHg in ICP (95% CI [0.06, 0.34], *P* = 0.004), while the 0.02 mmHg (95% CI [-0.03, 0.06], *P* = 0.423) increase in non-obese patients was not statistically significant. After adjusting for the confounders, a significant relationship between PEEP and ICP persisted in obese group (95% CI [0.05, 0.33], *P* = 0.007). The beta estimate coefficient of the effect of PEEP on ICP in the multivariable GEE model were displayed in Fig. 2. Univariate analyses indicated that PEEP was not associated with CPP (*P* = 0.452). But after adjusting for confounders, the relationship between PEEP and CPP (95% CI [-0.29, -0.01], *P* = 0.036) was statistically significant. The univariate analysis for the effect of PEEP on ICP and CPP in obese and non-obese patients were displayed in Fig. 3, respectively.

**Table 3:** Unadjusted and adjusted effect of PEEP on ICP and CPP

Variables	Unadjusted Analyses		Adjusted Analyses	
	PEEP beta estimate (95% CI)	*P* value	PEEP beta estimate (95% CI)	*P* value
ICP				
obese	0.2 (0.06, 0.34)	0.004	0.19 (0.05, 0.33)	0.007
non-obese	0.02 (-0.03, 0.06)	0.423	0.02 (-0.03, 0.06)	0.445
no LI	0.04 (-0.02, 0.1)	0.168	0.04 (-0.02, 0.1)	0.165
mild LI	0.17 (0.01, 0.33)	0.035	0.16 (0.01, 0.32)	0.038
moderate LI	0 (-0.24, 0.23)	0.974	-0.02 (-0.24, 0.19)	0.832
severe LI	0.02 (-0.05, 0.09)	0.586	0.02 (-0.05, 0.09)	0.614
TBI	-0.03 (-0.09, 0.04)	0.637	-0.03 (-0.08, 0.03)	0.529
non-TBI	0.01 (-0.07, 0.09)	0.539	0.01 (-0.06, 0.08)	0.458
obese + no LI	0.17 (0.02, 0.32)	0.029	0.17 (0.01, 0.33)	0.036
obese + mild LI	0.1 (-0.14, 0.34)	0.414	0.1 (-0.14, 0.35)	0.421
obese + moderate LI	0.29 (-0.11, 0.69)	0.162	0.23 (-0.14, 0.61)	0.216
obese + severe LI	0.23 (-0.11, 0.57)	0.179	-0.02 (-0.64, 0.6)	0.950
non-obese + no LI	0.03 (-0.03, 0.08)	0.354	0.03 (-0.03, 0.08)	0.335
non-obese + mild LI	0.21 (-0.01, 0.44)	0.063	0.18 (-0.03, 0.39)	0.093
non-obese + moderate LI	-0.17 (-0.43, 0.1)	0.213	-0.16 (-0.39, 0.07)	0.183
non-obese + severe LI	0.01 (-0.07, 0.09)	0.758	0 (-0.07, 0.08)	0.919
total	0.04 (-0.003, 0.09)	0.064	0.04 (-0.004, 0.09)	0.071
CPP				
obese	-0.12 (-0.44, 0.2)	0.452	-0.15 (-0.29, -0.01)	0.036
non-obese	-0.09 (-0.21, 0.04)	0.193	-0.01 (-0.07, 0.05)	0.717
no LI	-0.01 (-0.13, 0.1)	0.827	0 (-0.07, 0.07)	0.997
mild LI	-0.22 (-0.57, 0.12)	0.208	-0.08 (-0.26, 0.1)	0.393
moderate LI	-0.26 (-0.84, 0.33)	0.391	0 (-0.22, 0.21)	0.988
severe LI	-0.18 (-0.28, -0.08)	< 0.001	-0.07 (-0.18, 0.03)	0.182
TBI	-0.11 (-0.32, 0.09)	0.193	-0.12 (-0.36, 0.12)	0.407
non-TBI	-0.29 (-0.72, 0.14)	0.262	-0.17 (-0.59, 0.25)	0.522
obese + no LI	0.05 (-0.36, 0.45)	0.819	-0.11 (-0.28, 0.06)	0.199
obese + mild LI	-0.49 (-1.03, 0.06)	0.080	-0.16 (-0.46, 0.13)	0.280
obese + moderate LI	-0.31 (-1.07, 0.45)	0.422	-0.13 (-0.49, 0.24)	0.508
obese + severe LI	-0.49 (-1.34, 0.36)	0.259	-0.18 (-0.76, 0.4)	0.542
non-obese + no LI	-0.02 (-0.15, 0.1)	0.741	0.02 (-0.06, 0.09)	0.675
non-obese + mild LI	-0.07 (-0.53, 0.39)	0.767	-0.1 (-0.33, 0.12)	0.358
non-obese + moderate LI	-0.22 (-1.03, 0.6)	0.602	0 (-0.23, 0.23)	0.983
non-obese + severe LI	-0.17 (-0.25, -0.08)	< 0.001	-0.06 (-0.16, 0.05)	0.274
total	-0.09 (-0.21, 0.03)	0.140	-0.03 (-0.09, 0.02)	0.257

Models are adjusted for age, GCS on admission, APACHE IV score, tidal volume, respiratory rate, plateau pressure, mean arterial blood pressure, PaCO_2_, receiving vasopressors, and mannitol or hypertonic saline

*CI *confidence interval, *CPP *cerebral perfusion pressure, *ICP *intracranial pressure, *LI *lung injury, *PEEP *positive end-expiratory pressure, *TBI* traumatic brain injury

## Discussion

The major finding of the study was that unlike non-obese SBI patients, there was a statistically significant relationship between PEEP and ICP in obese SBI patients. However, the increase in ICP was tiny when compared with the applied PEEP level. The result could be translated as, in obese SBI patients, a 5 cmH_2_O increase in PEEP may potentially increase ICP by 1 mmHg by keeping all other covariates constant, which seemed be clinically inconsequential. The result suggested that when considering the effect of PEEP on ICP, obesity status may be an important factor.

The effect of PEEP on ICP had been reported by several investigators; however, there was no any definite consensus. Overall, three kinds of effect of PEEP on ICP in patients with brain injury had been reported, PEEP increasing ICP [13, 25–33], nonsignificant relationship between PEEP and ICP [15, 34, 35], other effects [36]. Recently, in a large retrospective study, Boone et al. found the application of PEEP for SBI patients with varying degrees of acute lung injury, made no clinically significant effect on ICP or CPP for overall patients; however, the effect may exist in the severe lung injury group [37]. The effect of PEEP on ICP may rely on several factors (lung recruitability, chest wall elastance, respiratory mechanics, intracranial compliance, baseline ICP, and et al.), and the results may be unchanged, increased, even decreased ICP according to PEEP increase [14]. Our study showed in overall SBI patients, PEEP had no significant impact on ICP; but in obese patients, a statistically significant relationship was found.

The effect of PEEP on ICP is ambiguous which may belong to two mainstreams: the lung and the brain. Increase in PEEP is transmitted to the pleural space, which in turn raises the central venous pressure (CVP). The elevation in CVP may reflect a reduced venous return which could increase ICP. Furthermore, elevated CVP could also directly increase ICP by decreasing jugular venous outflow [14]. The cranial ‘Starling resistor’ seemed to play an important role in the effect of PEEP on ICP [38]. Three key links were found between PEEP and ICP, the first link was the impact of PEEP on pleural pressure, the second link was the pleural pressure on CVP, and the third was the transmission of CVP to the internal jugular vein [14]. The transmission of PEEP to the pleural space depends on the relative elastance of the lung and the chest wall [18]. Compliant lung and/or stiff chest wall could make the transmission more effective, while decreased lung compliance may buffer the effect [15, 19, 20, 26]. In the present study, the severity of lung injury was not predictive of the effect of PEEP on ICP, only the mild lung injury was statistically significant, which may reflect the inhomogeneity of lung injury or may have other confounders that we were unable to measure. On the other hand, we defined the severity of lung injury only by the P/F ratio, which may not exactly reflect the lung injury.

Obesity is common in ICU and appears to be associated with lower mortality (‘obesity paradox’) [1, 39]. Previous studies showed respiratory elastance, including both the lung and the chest wall, tended to increase in obese patients with mechanical ventilation [6–8]. Obese patients had higher chest wall elastance. Stiffer chest wall may make the transmission of pressure more effective. In the present study, we found there was a statistically significant relationship between PEEP and ICP in obese SBI patients not for the non-obese ones. The results were in line with previous studies. However, the observed changes were relatively small, and this tiny increase in ICP may be clinically inconsequential.

The results of the current study had some clinical implications. It suggested that the effect of PEEP on ICP may differ between obese and non-obese SBI patients, which supported the significance of personalized strategy when considering the effect of PEEP on ICP. Future study could focus on the relationship of respiratory mechanics and ICP as well as the mediator effect of central venous pressure.

There were several limitations deserved to be mentioned. First, the retrospective design was subject to the inherent limitations. Residual confounders could influence the findings, although we attempted to account for this through multivariable models. A prospective study will be needed to confirm the findings. Second, the population was divided into obese and non-obese groups only according to BMI, without considering the fat distribution, which seemed to be relevant for chest wall elastance. Third, body weight was extracted on ICU admission, fluids balance could affect the body weight and then the BMI. Fourth, the present study included patients with ICP monitoring as well as mechanical ventilation, which represented a broad range of neurologic diagnoses. As for SBI patients, some of them had chest or abdominal trauma simultaneously, which could also influence the elastance of the chest wall. Fifth, because of the heterogeneity of different diseases, it may be difficult to generalize the results to patients with one particular neurologic diagnosis. Finally, the type and position of the ICP monitor were not recorded in the database, both of which may have an influence on ICP monitoring.

## Conclusion

Contrary to non-obese SBI patients, the application of PEEP for obese patients was associated with increased ICP. However, the effect of PEEP on ICP is modest and may be clinically inconsequential.

## Data Availability

Data analyzed during the present study are currently stored in the eICU database (eicu-crd.mit.edu).
